# Supplementary material for: Descriptive Epidemiology of breast and gynecological cancers among patients attending Saint Paul’s Hospital Millennium Medical College, Ethiopia
Source: PLoS One. 2020 Mar 20;15(3):e0230625. doi: 10.1371/journal.pone.0230625 (PMC7083302; doi:10.1371/journal.pone.0230625)
Supplement: Attachment — Submitted filename: Response to reviewers.docx [file pone.0230625.s002.docx]

**Response to reviewers**

Dear Dr Pirkko L. Härkönen,

Thank you very much for reviewing our manuscript. We also greatly appreciate the reviewers for their complimentary comments and suggestions. We have considered the comments that the reviewers suggested and revised the manuscript accordingly.

Please find attached a point-by-point response to reviewer’s concerns. We hope that you find our responses satisfactory and that the manuscript is now acceptable for publication.

Sincerely,

Haimanot Ewnetu Hailu

Assistant Professor of Epidemiology, Department of Public Health
Saint Paul's Hospital Millennium Medical College,AA, Ethiopia

Alternative Email: [haimanot.ewnetu@sphmmc.edu.et](mailto:hewnetu@yahoo.com)

Phone: +251979248856 / +251911983013

We appreciate that the reviewers’ comments. The followings are our point-by-point responses:

1. Is the manuscript technically sounds, and do the data support the conclusions?

**Response:** We believe we have followed scientifically sound methods to support our conclusion. We included all clients diagnosed to have gynecological and breast cancers, visited Oncology unit of SPHMMC from 2014 -2018 and who fulfill the inclusion and exclusion criteria. We didn’t calculate sample size because we aimed to see and include patients who visited this specific

Hospital within these five years period that was helpful to see the trend of this group of cancer. Our specific objectives were to determine the prevalence, pattern and trend of breast and gynecologic cancers and we made the conclusion based on our findings. The study used secondary data extracted from the patients’ chart and conducting an experiment was beyond the scope and objective of the study.

2. Has the statistical analysis been performed appropriately and rigorously?

**Response:** Since our study is aimed at characterizing the above mentioned group of malignancies, we used the appropriate method of descriptive analysis such as frequency distribution.

3. Have the authors made all data underlying the findings in their manuscript fully available?

**Response:** We have made the all the data underlying the findings available. The authors can make the additional data available up on request rather than having it deposited to a public repository. This is due to the fact that the data is accessed from the record of the patients which is taken from the hospital record/patient chart that needs to be kept confidential and only used for research purposes.

4. Is the manuscript presented in an intelligible fashion and written in standard English?

**Response:** The fact that the two of the authors’ mother tongue is English makes it easier to resolve the issues associated with language standard and all the authorsa edited and made maximum effort to improve the language quality. This issue is addressed in the manuscript accordingly.

**5. Review Comments to the Author**

**Reviewer #1**

We appreciate the reviewer’s comments. The followings are our point-by-point responses:

1. The manuscript has been review and found to have merit for publication. The authors should address the issues identified and re-submit:
   Gynecological cancer in the title should read: "gynecological cancers" instead of cancer. In figure 1, cervical cancer which has the highest frequency was omitted. Kindly add that of cervical cancer cases. Secondly the title for figure 1 is not appropriate as these measures presented are not prevalence but rather proportional morbidity of breast and gynecological cancers presenting at SPHMMC. Change the title of the figure and write the appropriate title for figure 1.
   **Response:** The comment is well accepted and addressed in the manuscript accordingly.

Proportion of breast and gynecologic cancers

| Tumor location | Frequency (%) |
| --- | --- |
| cervix | 46.7 |
| breast | 29.3 |
| Ovary | 12.9 |
| Endometrium | 6.2 |
| Myometrium | 2.4 |
| Others | 1.6 |
| FT&BL | 0.6 |

Figure1: Proportional morbidity of breast and gynecological cancers among clients presenting at SPHMMC, 2014-18
2. Figure 2, the line graph used for presentation is not appropriate. The author should used a multi-bar graph to show the age distribution since this is not a time trend but rather age distribution. The title should change to the age distribution of breast and gynecological cancers presenting at SPHMMC, 2014 – 2018

**Response:** The comment is well accepted and addressed accordingly.

Age distribution of breast and gynecological cancers

Frequency (No)

| Age groups | breast | Cervix | Ovary | Endometrium | FT & BL | Myometrium | others |
| --- | --- | --- | --- | --- | --- | --- | --- |
| 15-19 | 3 | 3 | 12 | 2 | 2 | 1 | 0 |
| 20-24 | 17 | 4 | 12 | 1 | 1 | 0 | 0 |
| 25-29 | 59 | 20 | 27 | 3 | 1 | 4 | 5 |
| 30-34 | 49 | 55 | 22 | 7 | 0 | 1 | 10 |
| 35-39 | 128 | 112 | 33 | 17 | 2 | 11 | 8 |
| 40-44 | 66 | 100 | 22 | 16 | 0 | 3 | 0 |
| 45-49 | 86 | 133 | 29 | 7 | 3 | 5 | 2 |
| 50-54 | 57 | 139 | 31 | 17 | 1 | 6 | 1 |
| 55-59 | 36 | 129 | 27 | 17 | 0 | 3 | 1 |
| 60-64 | 36 | 113 | 23 | 18 | 2 | 5 | 2 |
| 65+ | 50 | 127 | 22 | 20 | 1 | 10 | 4 |

Figure 2: Age distribution of breast and gynecological cancers presenting at SPHMMC, 2014 - 2018

2. There is no table or figure to show the distribution of the cases by place. I suggest the authors show the distribution by place using a spot map or any other appropriate presentation. **Response:** Thank you. The comment is accepted and well addressed in the manuscript. We prefer to present the information using table because we only have information of patients that came to seek help from SPHMMC and the number of cases that has been visited in other facilities is beyond the objective of the study and are not included.

Table1: Distribution of breast and gynecological cancers presenting at SPHMMC by place of residence 2014-18

| **Place of residence** | **Frequency No (%)** |
| --- | --- |
| Addis Ababa | 1098(54.8%) |
| Oromia | 763(38.1%) |
| Amhara | 126(6.3%) |
| Tigrai | 15(0.75) |

3. The authors indicated that, by 2028, the numbers of new cases are expected to double from 2018, increasing from 554 new cases to 1141 new cases per year (Figure 4). However the calculation or model used to arrive at that is not shown in the methods or results. The authors should clearly show the methodology and assumption in arriving at that projected figure.
   **Response:** Thank you for the comment. It is well addressed both in methods and results section. We have projected the number of cancer cases for the next ten years using method of best fit (least squares) linear regression. The basis for our forecast was the number cases observed from 2014-18.
4. The authors also indicated in the last paragraph of the results that, the association between the number of new cases and time was marginally statistically significant (P=0.054). I suggest this statement should be deleted as the p-value of 0.054 is not significant and that statement add nothing relevant to the results.
   **Response:** Comment well received and corrected (deleted) accordingly.

**Reviewer #2**

We appreciate the reviewer’s comments. The followings are our point-by-point responses:

1. It helps the reviewer, if page and line numbers would be placed

**Response:** Comment well accepted.
2. Demographic patterns: As to Oromia and Amhara regions, do the figures of lower numbers of cancer cases reflect the distance to the hospital or rather a lower population.
**Response:** Thank you for your comment. The figures of lower number cases reflect both the distance from the hospital and lower number of cases visiting the study hospital (SPHMMC). The reason we have mentioned this is that majority of the patients are from Addis ababa which is where the hospital is located and Oromia is a region bordering Addis Ababa. Majority of the patients are from Oromia, next to Addisbaba, followed by Amhara which is proportional to the distance of the regions from the SPHMMC hospital and lower number of cases visiting the facility due to the possible distance of the facility from their place of residence.

3. Time trends: does the increase of cancer cases mean a real increase of cases or possibly better diagnostics and increased remittance.
**Response:** Thank you for your comment. As you have mentioned, the possible explanation for increased number of cases is either real increase in incidence of disease or availability of better diagnostic modality, increased patient awareness etc. and we have, mentioned this possibilities in the discussion section of this manuscript. Even though this study is purely descriptive, different literatures are reporting the increment of number of cases of non communicable disease including cancer in developing countries including Ethiopia and we believe the increase in number of new cases of cancer is more plausible in addition to the above mentioned explanations.

4. Discussion: I do not understand, how the early marriage or bigger number of life births explain the high nb of cases of cervical cancer. Cervical cancer is a sexually transmitted infection (HP-virus) which is almost always involved in the development of cervical cancer. The possibility for transmission depends on nb of intercourse without condom, nb of partners etc.

**Response:** Thank you for your comment. You are right but we have been thinking of how being exposed to sexual intercourse in the early age is a risk factor for having multiple sexual partner which is a risk factor for any STI including HPV and subsequent Cervical cancer. Now we tried to elaborate what we mean by this statement in the discussion section.

5. I do not understand what environmental factors you mean.
Last page: "Breast cancer and gynecological cancers are largely preventable diseases..." Cases of breast cancer can largely be prevented with mastectomies (in those 5-10% of women who have hereditary BRCA1 or 2 genes). Cervical cancer can be prevented by rigorous use of condoms or a vaccination before initiation of coital activities. The other gynecological cancers can not be prevented, except by surgically removing the gyn organs. Instead, with good early diagnostics and adequate forms of therapies, the number of deaths due to cancers can be prevented.

**Response:** Thank you for your comment, its well understood. In this case we are trying to refer the general(non specific) prevention strategies like life style changes related to diet, physical activity, alcohol drinking, smoking etc. and early screening. [Grimes DA](https://www.ncbi.nlm.nih.gov/pubmed/?term=Grimes%20DA%5BAuthor%5D&cauthor=true&cauthor_uid=7847546), [Economy KE](https://www.ncbi.nlm.nih.gov/pubmed/?term=Economy%20KE%5BAuthor%5D&cauthor=true&cauthor_uid=7847546). Primary prevention of gynecologic cancers. [Am J Obstet Gynecol.](https://www.ncbi.nlm.nih.gov/pubmed/7847546) 1995 Jan;172(1 Pt 1):227-35
