# Supplementary material for: Descriptive Epidemiology of breast and gynecological cancers among patients attending Saint Paul’s Hospital Millennium Medical College, Ethiopia
Source: PLoS One. 2020 Mar 20;15(3):e0230625. doi: 10.1371/journal.pone.0230625 (PMC7083302; doi:10.1371/journal.pone.0230625)
Supplement: Attachment — Submitted filename: Response to reviewers.docx [file pone.0230625.s003.docx]

**Response to reviewers**

Dear Dr Pirkko L. Härkönen,

Thank you very much for reviewing our manuscript. We also greatly appreciate the reviewer for his/her complimentary comments and suggestions. We have considered the comments that the reviewer suggested and revised the manuscript accordingly.

Please find attached a point-by-point response to reviewer’s concerns. We hope that you find our responses satisfactory and that the manuscript is now acceptable for publication.

Sincerely,

Haimanot Ewnetu Hailu

Assistant Professor of Epidemiology, Department of Public Health
Saint Paul's Hospital Millennium Medical College,AA, Ethiopia

Alternative Email: [haimanot.ewnetu@sphmmc.edu.et](mailto:hewnetu@yahoo.com)

Phone: +251979248856 / +251911983013

We appreciate the reviewer’s comments. The followings are our point-by-point responses:

**Reviewer 2**

5. I do not understand what environmental factors you mean.
Last page: "Breast cancer and gynecological cancers are largely preventable diseases..." Cases of breast cancer can largely be prevented with mastectomies (in those 5-10% of women who have hereditary BRCA1 or 2 genes). Cervical cancer can be prevented by rigorous use of condoms or a vaccination before initiation of coital activities. The other gynecological cancers can not be prevented, except by surgically removing the gyn organs. Instead, with good early diagnostics and adequate forms of therapies, the number of deaths due to cancers can be prevented.

**Response:** Thank you for your comment, its well understood. We have reconsidered and restated the sentence as follows. “General, nonspecific prevention mechanisms including change in life styles and early screening would help to minimize risk of majority of breast and gynecological cancers. However, specific measures like mastectomy may prevent breast cancer while cervical cancer can be prevented with safe sexual practices and vaccination.”
